# Climate Change and AMR: Interconnected Threats and One Health Solutions

**DOI:** 10.3390/antibiotics14090946

**Published:** 2025-09-18

**Authors:** Bilal Aslam, Sulaiman F. Aljasir

**Affiliations:** Department of Veterinary Preventive Medicine, College of Veterinary Medicine, Qassim University, Buraydah 51452, Saudi Arabia; b.aslam@qu.edu.sa

**Keywords:** antimicrobial resistance (AMR), climate change, One Health, global health, infectious disease, antibiotics, environment

## Abstract

Climate change is a significant driver of antimicrobial resistance (AMR) and infectious disease dynamics, presenting urgent and interconnected global health challenges. Rising temperatures, ecosystem alterations, and extreme weather events amplify the global spread of resistant pathogens, zoonotic infections, and vector-borne diseases. These impacts disproportionately affect low- and middle-income countries (LMICs), escalating healthcare costs and straining limited infrastructure. A critical characteristic of bacterial resistance is that it often does not incur a fitness cost, underscoring the necessity of preventive strategies to mitigate climate-driven AMR emergence, rather than relying on reactive treatments after resistance is established. Climate change accelerates AMR primarily by increasing the prevalence of infectious diseases, which in turn drive higher antibiotic use and select resistance. The socioeconomic consequences are particularly severe in LMICs, where high climate vulnerability converges with weaker health systems. Pandemic-related disruptions provided key insights into environmental dynamics, with notable temporary reductions in nitrogen dioxide (NO_2_) emissions, i.e., 20–30% in China, Italy, France, and Spain, and approximately 30% in the USA, which highlights the responsiveness of ecosystems to human activity. Unlike prior reviews that treated AMR and climate change as separate issues, this article integrates mechanistic evidence, epidemiological insights, and global strategies to provide a comprehensive One Health framework addressing these synergistic threats. We conclude that AMR and climate change are interlinked crises requiring urgent, integrated interventions. The quadripartite (FAO, UNEP, WHO, WOAH) provides a crucial framework for the coordinated cross-sectoral strategies, strengthened surveillance, and robust antibiotic stewardship required to mitigate this dual threat and safeguard global health security.

## 1. Introduction

Rapid developments in technology, industry, and research are posing serious challenges to planetary ecosystems and global health. Among the most critical of these interconnected threats are climate change and antimicrobial resistance (AMR). Both crises are ultimately driven by anthropogenic choices and lifestyles. The One Health concept, which highlights the intrinsic connections between human health, animal health, and their shared environments, provides a vital framework for addressing these issues and is central to future sustainability [1]. Climate change refers to significant long-term statistical shifts in climatic variables, impacting sensitive factors such as temperature extremes, precipitation patterns, wind, and humidity [2]. While the mean temperature of earth has remained relatively stable over millennia, anthropogenic activities have caused a rapid 1 °C rise in global temperatures in recent decades. This shift has altered precipitation, increased the frequency of extreme weather events, and disrupted both aquatic and terrestrial ecosystems [3,4]. This anthropogenic climate catastrophe endangers millions of species and is directly linked to increased human morbidity and mortality, underscoring the urgent need to reduce the substantial environmental footprint of the healthcare sector itself [5]. The Intergovernmental Panel on Climate Change (IPCC) forecasts a global temperature increase of 1.5 °C to 5.8 °C in the 21st century, alongside a rise in extreme weather events such as hurricanes and droughts. They emphasize that even minor climatic shifts can jeopardize human health in multiple ways [6].

Microorganisms, as the earliest forms of life, possess sophisticated adaptation mechanisms including gene transfer, biofilm development, and quorum sensing that allow for rapid evolution and resilience in the face of environmental change [7]. Numerous studies indicate that climate change can favor infectious diseases, with rising temperatures influencing the geographic distribution of pathogens and increasing the incidence of skin-related and other climate-sensitive illnesses [8].

The disease burden typically quantified through metrics such as disability-adjusted life years (DALYs), years of life lost (YLLs), mortality, and healthcare costs provides a framework for assessing the impact of both climate-sensitive diseases and AMR [9]. Explicitly defining this burden is essential for comparing health threats and highlighting the overlapping vulnerabilities posed by these dual crises. For instance, climate-sensitive infections like salmonellosis and malaria contribute significantly to global morbidity, while drug-resistant pathogens are already responsible for nearly five million deaths annually. Recognizing these parallel burdens strengthens the rationale for integrated assessments under the One Health approach.

The health consequences of climate change are severe and multifaceted, including heat-related mortality, food insecurity, decreased crop yields, sea-level rise, cardiovascular morbidity, increased susceptibility to infections, and fatalities from wildfires [10]. Even a 1 °C rise in ambient temperature may lead to a 5–10% increase in salmonellosis cases, which already causes over a million foodborne infections annually in the United States alone [11]. This could translate to an extra 50,000–100,000 cases and 27,000 hospital admissions per year for this single disease in one region. Over 200 diseases are spread through contaminated food and water, many of which require antibiotic therapy. Therefore, by increasing the incidence of these diseases, climate change has substantial potential to indirectly drive antibiotic use and resistance [12].

Poor infection prevention and control (IPC) strategies further facilitate the spread of bacteria, which can develop multiple resistance mechanisms, limit treatment options, and support the rapid dissemination of AMR organisms in the environment [13]. AMR is a dire global problem, currently causing an estimated 700,000 deaths per year, with projections suggesting severe economic implications and healthcare costs reaching $300 billion to $1 trillion by 2050 [14]. While antimicrobial agents are vital to human medicine, agriculture, and aquaculture for treating diseases and ensuring food security, their misuse is a primary driver of resistance. Hospitals, farms, pharmaceutical effluent, wastewater treatment plants, and international travel all serve as critical reservoirs and pathways for the dispersal of resistance genes [15].

In 2019, drug-resistant infections were associated with nearly 5 million deaths, with 1.27 million directly attributable to AMR; the greatest burden falls on low-resource nations. Nearly one million of these deaths were linked to six major pathogens, i.e., *Escherichia coli*, *Staphylococcus aureus*, *Klebsiella pneumoniae*, *Streptococcus pneumoniae*, *Acinetobacter baumannii*, and *Pseudomonas aeruginosa* particularly when exhibiting multidrug resistance [16]. The recent discovery of plasmid-mediated colistin resistance (*mcr* genes) exemplifies the rapid evolution of the AMR crisis. Colistin, a last-resort antibiotic, has seen its efficacy rapidly eroded by mobile genetic elements that spread swiftly across bacterial populations and global regions. The *mcr-1* gene, first identified in *E. coli*, has now been detected worldwide, posing a critical challenge for treating multidrug-resistant Gram-negative infections [17].

Researchers have recognized that the parallels between climate change and AMR extend beyond their shared attributes of urgency, severity, and global reach. These crises are also mechanistically linked, as climate change can directly exacerbate AMR, for instance, by expanding the range and activity of disease vectors [18]. From an individual perspective, the use of antimicrobials and fossil fuels is rationalized by their immediate benefits: antibiotics effectively treat infections, while fossil fuels provide inexpensive and convenient energy for daily life. However, the public is often less aware of the adverse long-term consequences, such as immune compromise or environmental degradation, that accompany these advantages [19].

Public perception plays a critical role in how societies respond to complex health challenges. A limited understanding of the long-term implications of climate change and AMR frequently results in delayed responses, inappropriate antibiotic use, and resistance to policy measures. In contrast, better-informed communities are more likely to support stewardship programs, adopt sustainable practices, and comply with public health initiatives [20]. Thus, understanding and shaping public opinion is essential for developing effective interventions.

Global disease burdens are increasingly affected by climate change, though the complexity of climate-dependent variables makes precise health impact assessments challenging. Similarly, while antimicrobials are vital for treating infections, their overuse and misuse have accelerated the emergence of resistance [21]. Due to the intricate nature of these challenges, the World Health Organization (WHO) identified both AMR and climate change as top ten global public health threats in 2019 [22].

Although climate change and AMR are recognized as critical global health issues demanding concrete solutions, insufficient research has explored their interconnections. Contemporary scholarship has predominantly examined these threats in isolation, with only a limited number of studies investigating how climatic factors such as increased temperatures, humidity, and extreme weather events accelerate the dissemination of resistant pathogens. This review addresses that gap by systematically integrating evidence from diverse fields, including environmental science, microbiology, and public health, within a unified One Health framework. A comprehensive literature review methodology is particularly suited to this objective, as it enables the synthesis of disparate findings, the identification of convergent mechanisms, and the formulation of policy-relevant insights. In doing so, this review aims to elucidate not only the connections between AMR and climate change but also how this understanding can inform more effective surveillance, stewardship, and global mitigation strategies.

## 2. Research Methodology

This article provides a comprehensive analysis of the current state of knowledge on the interconnected threats of climate change and AMR through a systematic literature review of peer-reviewed publications. This methodology facilitates the identification of knowledge gaps, the synthesis of insightful information, and the formulation of well-informed conclusions, ensuring a rigorous overview of existing research.

A search was conducted using Google Scholar to identify relevant literature. Key search terms included “public health,” “disease surveillance,” “artificial intelligence,” “antimicrobial resistance,” “climate change,” “One Health,” and various combinations thereof. The search and selection process adhered to the Preferred Reporting Items for Systematic Reviews and Meta-Analyses (PRISMA) guidelines to ensure transparency and reproducibility.

**Figure 1:**
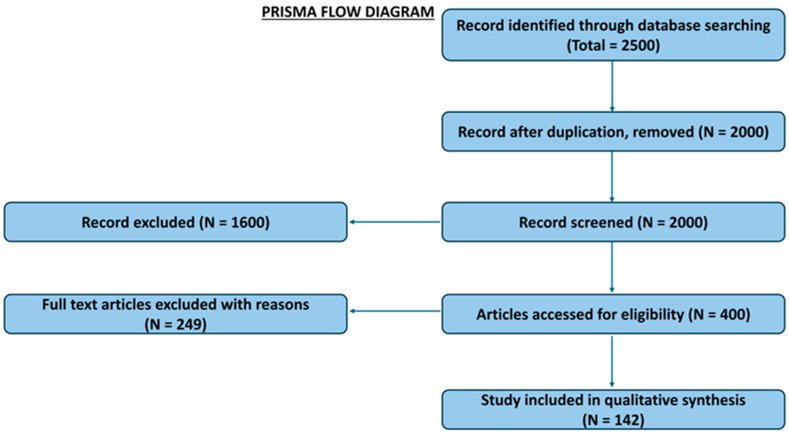
PRISMA flow diagram illustrating study selection process.

The present review is based on an analysis of scientific papers examining the relationship between antimicrobial resistance and climate change. The included publications encompass a range of topics, such as the influence of climate change on AMR and infectious disease dynamics, the associated socioeconomic impacts, connections to food safety and the COVID-19 pandemic, and relevant global strategies. A final manual filtering process involved selecting the most relevant publications and performing a backward and forward citation search of their bibliographies to identify additional sources. Following this rigorous screening process, a final list of 142 publications, all containing data essential for this review, was compiled. The detailed selection process is summarized in the PRISMA flow diagram (Figure 1).

## 3. Climate Change and Public Health

Health is a fundamental component of human well-being, profoundly influencing happiness, quality of life, economic productivity, and societal growth. Health outcomes are shaped by a complex interplay of factors, including socioeconomic status, race, gender, age, pre-existing medical conditions, genetics, occupation, and geographic location [23]. Lifestyle choices such as diet, physical activity, and substance use along with social support networks and access to care, also play a critical role in determining health trajectories [24].

**Figure 2:**
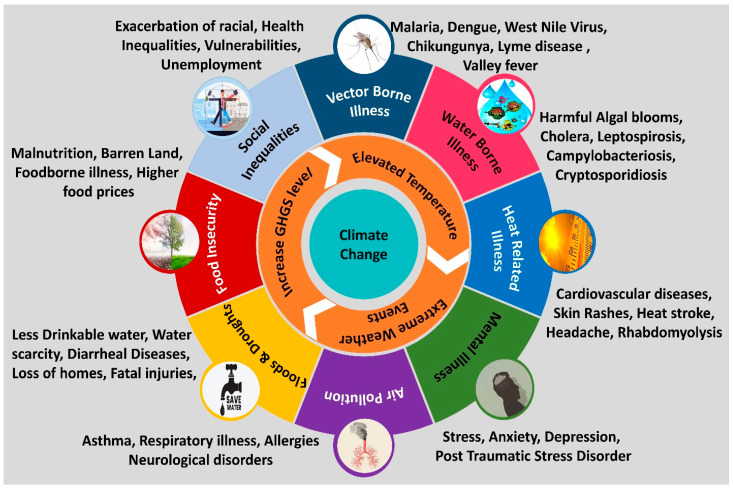
Impacts of Climate Change on Global Health.

Robust healthcare systems, strong social networks, and community support are essential for fostering resilience and positive health outcomes. However, these foundations are increasingly threatened by climate change. The climate crisis is amplifying the frequency and intensity of extreme weather events including heatwaves, floods, droughts, storms, and wildfires which pose direct and immediate risks to human life. Furthermore, climate change is altering the distribution and behavior of disease vectors, leading to a heightened incidence of vector-borne diseases such as malaria, dengue, Lyme disease, and Zika virus. Shifts in temperature and precipitation patterns also degrade air and water quality and compromise food security, with severe implications for public health [24]. Concurrently, the mental health burden associated with climate change encompassing stress, anxiety, depression, and trauma is growing, further challenging individual and community resilience [23]. A summary of the multifaceted health impacts of climate change is presented in Figure 2.

Heat-related illness: Rising global temperatures and more frequent, intense heatwaves are driving an increase in heat-related illnesses, representing one of the most direct consequences of climate change on human health. Conditions such as heat stroke, exhaustion, cramps, and rashes can be severe or fatal, particularly among vulnerable populations including infants, young children, the elderly, outdoor workers, and individuals with pre-existing medical conditions [25]. In the United States, extreme heat events pose a significant public health risk. Metropolitan areas such as St. Louis, Philadelphia, Chicago, and Cincinnati have reported increased mortality attributable to heat stroke, cardiovascular, respiratory, and cerebrovascular disorders [26]. Climate models project that such extreme heat events will become more widespread and severe in the coming decades. Although the risks of heat-related illness and death have declined in recent years likely due to improved forecasting, heat-health early warning systems, and greater access to air conditioning. However, this progress may be offset by growing vulnerabilities. Factors such as urban heat islands, aging demographics, and increased urbanization are expected to heighten population susceptibility to heat-related health impacts. It is also noted that milder winters resulting from a warmer climate may reduce cold-related morbidity, injuries, and fatalities. However, these potential benefits are unlikely to compensate for the projected increase in heat-related mortality [27].

Infectious diseases: Climate change significantly alters the transmission dynamics of infectious diseases, posing a substantial threat to global public health (Table 1). Rising temperatures expand the geographic range and prolong the seasonal activity of disease vectors such as mosquitoes and ticks, enabling them to establish populations in previously unaffected areas. Warmer conditions can accelerate the life cycle of mosquitoes, increasing their reproduction rates and shortening the incubation period for pathogens like those causing malaria and dengue fever [28]. Changes in precipitation patterns further modulate disease spread. Increased rainfall and flooding can contaminate water sources, facilitating the transmission of waterborne pathogens such as *Vibrio cholerae* and *Salmonella* Typhi. These risks are exacerbated in regions with poor sanitation infrastructure and limited access to clean water, disproportionately affecting vulnerable communities. Furthermore, severe weather events including hurricanes, floods, and droughts could disrupt ecosystems, alter landscapes, and displace populations. The resulting overcrowding in temporary shelters, coupled with compromised sanitation and limited healthcare access, creates ideal conditions for disease outbreaks [27].

Critically, the increased incidence of infectious diseases driven by climate change has a consequential, indirect effect on AMR. The expansion of mosquito vectors’ range and breeding season intensifies the transmission of diseases like malaria and dengue, which in turn leads to greater and often inappropriate use of antimicrobials for treatment and prophylaxis [38]. Similarly, climate-driven events, such as drought-induced movements of bat populations, have been linked to zoonotic spillover, including the emergence of coronaviruses like SARS-CoV-2 [39]. These examples illustrate a vital pathway through which climatic disturbances alter infectious disease ecology, thereby amplifying the burden of antibiotic resistance.

Deteriorating air quality: Climate change is projected to exacerbate ground-level ozone and particulate matter pollution in many regions, leading to significant adverse health outcomes. These include compromised lung function, increased hospitalization and emergency room visits for asthma and allergies, and a rise in premature mortality. The formation of ozone is influenced by multiple climate-sensitive factors, including rising temperatures, concentrations of precursor chemicals, methane emissions, wildfire smoke, and air stagnation events. As global temperatures increase, the associated intensification of air pollution is likely to result in a greater number of premature deaths. Regional estimates suggest that by 2050, the combined health impacts of ozone and particulate matter could cause an additional 1000 to 4300 premature deaths annually. The economic burden of this pollution is substantial; a U.S. review of the health implications from ozone levels between 2000 and 2002 estimated the health-related costs of ozone levels exceeding national guidelines at $6.5 billion nationwide [40].

Mental health impacts: Beyond its environmental consequences, climate change exerts profound and multifaceted effects on mental health. The experience of ecological degradation and extreme weather events can trigger eco-anxiety, grief, and significant psychological distress, including post-traumatic stress disorder (PTSD), depression, and anxiety. This ecological grief often stems from the perceived loss of biodiversity, ecosystem stability, and a familiar environment [28]. Climate-related stressors such as extreme heat, air pollution, and forced displacement are also associated with increased levels of stress, interpersonal hostility, and violence. Moreover, feelings of nihilism, powerlessness, and pessimism about the future are common, which can foster hopelessness, impede proactive engagement, and exacerbate depressive disorders. In the aftermath of catastrophic weather disasters, trauma and PTSD are prevalent, with children and other vulnerable groups being disproportionately affected. For Indigenous and local communities, environmental losses pose a particularly severe threat to cultural identity, traditional knowledge, and social cohesion [41].

Furthermore, climatic factors like urban heat islands and elevated air pollution levels have been linked to reduced cognitive function and an increased risk of neurodegenerative diseases such as dementia. The resulting mental and physical health conditions carry significant consequences for individuals, communities, and economies. They can adversely affect social relationships, limit employment opportunities, and hinder economic development. For businesses, these health issues translate into substantial costs that amounts to billions of dollars annually due to absenteeism, lost productivity, and increased healthcare expenditures [42].

Public health organizations must prioritize recognizing these psychological impacts to effectively identify at-risk populations, expand access to mental health services, raise public awareness, and implement resilience-building interventions. Fostering collective psychological adaptability is essential to safeguarding community mental health in the face of ongoing climate change [43].

Uneven health impacts: Climate change exacerbates existing social and economic disparities, disproportionately burdening underserved and vulnerable populations. During extreme heat events, low-income households are often confined to outdated or substandard housing with inadequate cooling infrastructure. A lack of financial resources and resilient infrastructure further impedes their ability to prepare for and recover from climate-related disasters, deepening their vulnerability [44].

These health disparities are compounded by financial constraints that limit access to health insurance and medical care. Indigenous communities, including Native American, Alaska Native, and Pacific Islander populations, are among the most severely affected. Climate change jeopardizes traditional food sources, exacerbating food insecurity and contributing to negative mental health outcomes. In Alaska, the melting permafrost damages critical infrastructure, leading to physical harm and community displacement. For tribes in remote or rural areas, climate-related flooding and drought can severely compromise access to clean water [42].

Communities of color and immigrant populations face additional challenges, including systemic discrimination, language barriers, and exclusion from urban and climate planning processes. This marginalization often results in neighborhoods that are ill-prepared for climate impacts and lack protective resources [44]. These same communities exhibit higher prevalence of chronic conditions such as asthma and heart disease, which are aggravated by climate factors including elevated temperatures and air pollution.

Furthermore, individuals with disabilities or chronic illnesses face acute risks during extreme weather events. Evacuations and access to safety are hampered by unreliable transportation, inaccessible emergency instructions, and interruptions to essential medical services and power-dependent medical equipment [45]. These compounding inequities underscore the critical need for fair, inclusive, and targeted climate adaptation policies that prioritize the needs of marginalized groups to ensure a just and equitable response to the climate crisis

## 4. AMR and Climate Change: Interlinked Global Challenges

Rising global temperatures, humidity, and precipitation patterns due to climate change are projected to facilitate the spread of bacterial pathogens, increase antibiotic consumption, and amplify the global burden of AMR [46]. A foundational US study demonstrated that a 10 °C increase in minimum temperature was associated with a significant rise in the proportion of antibiotic-resistant strains: 4.2% for *E. coli*, 2.2% for *K. pneumoniae*, and 2.7% for *S. aureus*. This finding indicates that climate-induced temperature increases directly promote antibiotic resistance in pathogenic bacteria, underscoring the necessity for further research into this environmental driver of AMR [47].

Beyond this direct correlation, multiple climate-sensitive mechanisms contribute to the proliferation of AMR. A 28-country analysis revealed that nations with a 10 °C higher mean ambient temperature experienced significantly faster increases in resistance rates for *E. coli* and *K. pneumoniae*, highlighting the substantial role of environmental factors in regulating AMR dynamics [48]. Furthermore, extreme weather events, particularly flooding, act as catalysts for the spread of resistant pathogens. For instance, the cholera outbreak in Yemen, driven by resistant *Vibrio cholerae* strains, was directly linked to flood-induced damage to sanitation infrastructure [49]. At a molecular level, laboratory evidence suggests that increased heat stress can stimulate horizontal gene transfer between bacteria, thereby accelerating the dissemination of resistance genes [50]. These findings collectively imply that climatic stressors not only expand the geographical range of infections but also actively expedite the genetic mechanisms that underpin resistance.

**Figure 3:**
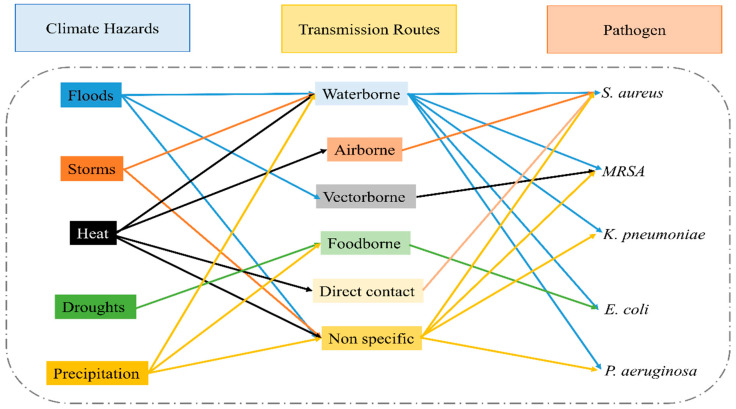
Climate hazards can significantly impact the transmission and health consequences of resistant pathogens.

The pathways through which climate change impacts human health are both direct and indirect. The current research has largely focused on direct effects such as injuries and mortality from heatwaves, floods, and storms [51] (Figure 3). However, a growing body of evidence illustrates profound indirect consequences. Climate change modulates infectious disease dynamics by altering pathogen distribution, vector reproduction rates, and transmission environments. These effects ultimately determine the shifting frequency, intensity, and seasonal patterns of disease outbreaks on a regional scale [52].

**Figure 4:**
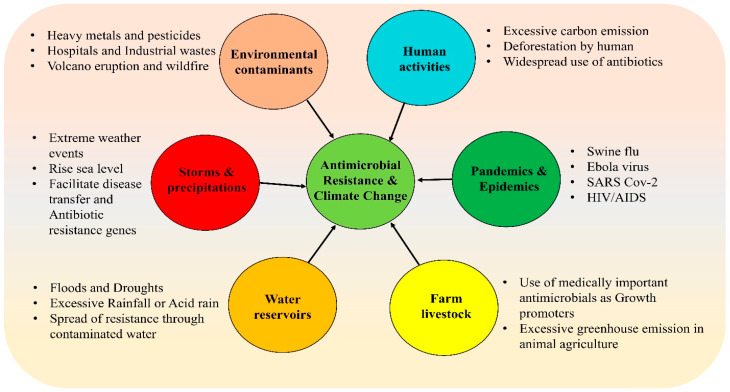
Factors involved in the relationship between antimicrobial resistance and climate change.

Over time, the health implications of climate change spanning physical, environmental, social, and behavioral dimensions continue to intensify [53]. This pattern of escalating risk is directly paralleled in the ongoing crisis of AMR. A deeper understanding of the co-evolution of these two global threats is critical for designing more effective and integrated future interventions [54]. The interconnected drivers and feedback loops linking climate change and AMR are illustrated in Figure 4.

Beyond increasing human-pathogen contact, climate change enhances several characteristics of pathogens themselves, including vector interaction efficiency, life cycle acceleration, reproductive fitness, and transmission duration. For instance, warming temperatures positively impact mosquito population growth, survival, biting rates, and viral replication, thereby increasing the transmission efficiency of pathogens like West Nile virus [55]. Concurrently, ocean warming accelerates the proliferation of harmful illnesses and toxic algal blooms caused by cyanobacteria, dinoflagellates, and *Pseudonitzschia* spp. [56]. Excessive precipitation can lower the pH of coastal waters, creating conditions that favor the abundance of *Vibrio cholerae* and *V. vulnificus* and increase the risk of cholera outbreaks [57].

These changes contribute to the emergence and spread of drug-resistant infections that are difficult or potentially impossible to treat with existing antimicrobials, including antibiotics, antivirals, antifungals, and antiparasitics. As a result, common bacterial infections, surgical and postpartum complications, fungal diseases, HIV, and malaria may become more deadly and harder to manage, potentially resulting in millions of additional lives lost annually. Addressing this dual threat requires overcoming significant societal and political challenges. Public misperception of health risks and disengagement from global issues complicates response efforts, while reducing antibiotic misuse remains particularly difficult in an era of expanding yet inequitable access to healthcare [58]. Policymakers must therefore develop innovative strategies to communicate the tangible realities of climate change and AMR to motivate public action. National and international action plans should prioritize integrated measures that explicitly address the interconnected nature of these two crises [59].

There is growing interest in establishing an Intergovernmental Panel on Climate Change (IPCC)-like body for AMR to centralize and accelerate global coordination [60]. Although 117 countries have developed AMR National Action Plans aligned with the Global Action Plan, concurrent global crises especially climate change threaten to undermine ongoing AMR mitigation efforts. The Global Leaders Group on Antimicrobial Resistance has emphasized the need for high-level political advocacy to ensure AMR is incorporated into high-level climate policy discussions [61].

Investments in managing and controlling both AMR and climate change are expected to yield significant returns, far outweighing the costs. Increased funding is urgently needed to integrate the climate–AMR nexus into existing One Health plans and initiatives. Furthermore, fostering international collaborative responses is essential to effectively manage these interconnected threats. Forecasting the future expansion of AMR is also critical for estimating the avoidable disease burden through interventions aimed at reducing antibiotic use and developing new antimicrobial agents [62].

### Burden of Diseases and Economic Impact of AMR in Context to Climate Change

AMR already imposes a significant global burden of disease, a crisis that climate change is poised to exacerbate. In 2019, AMR was associated with an estimated 4.95 million deaths worldwide, with 1.27 million deaths directly attributable to resistant infections caused by pathogens such as *E. coli*, *S. aureus*, and *K. pneumoniae* [63]. As climate change amplifies environmental conditions that favor the spread of infectious diseases and the selection for resistance, healthcare systems will face escalating costs associated with treating increasingly complex infections. The World Bank estimates that, by 2030, AMR could reduce global annual GDP by up to US $3.4 trillion, with the most severe economic impacts felt in low- and middle-income countries (LMICs) [14]. These costs encompass not only direct healthcare expenditures but also productivity losses, increased food insecurity, and disruptions to trade and livelihoods [64].

However, the economic modeling of AMR’s burden involves considerable uncertainty. Some analyses question the precision of current estimates, noting that they often rely on limited evidence and shaky assumptions regarding transmission dynamics and attributable mortality [65]. Despite these uncertainties, the potential scale of the crisis is und vast. Assessments commissioned by the UK government suggest that, if left unaddressed, AMR could cause up to 10 million annual deaths globally by 2050, trigger a 2–3.5% reduction in GDP, and accumulate costs of up to $100 trillion [66].

Climate change independently threatens economic stability and development. A 2021 report by the Swiss Re Institute projected that climate change could reduce global economic output by 11–14% up to $23 trillion annually by 2050. The economic consequences will be profoundly unequal. While high-income countries may face significant losses (e.g., an estimated 7% GDP reduction in the United States), many low-income countries could experience devastating economic declines of 20% or more, despite having contributed least to greenhouse gas emissions [67,68]. This injustice is mirrored in the health impacts of AMR, where LMICs, which are also most vulnerable to climate-sensitive infectious diseases, face a disproportionate double burden.

Climate change magnifies the AMR burden through several key pathways. Higher ambient temperatures are correlated with increased resistance rates. One multi-country study found that a 10 °C rise in mean ambient temperature was associated with a 4.2% increase in resistance in *E. coli* and a 2.2% increase in *K. pneumoniae* [69,70,71]. Floods and droughts facilitate the spread of resistant pathogens in water and food systems, leading to higher infection rates and increased antimicrobial usage [72]. Warming scenarios project a rise in foodborne illnesses like salmonellosis and waterborne diseases like cholera, indirectly driving up antimicrobial consumption and thus resistance emergence [73].

The consequences extend into clinical practice as well: declining efficacy of antibiotic prophylaxis could lead to significantly more surgical site infections and related deaths, imposing additional costs on health systems. Given these overlapping challenges, the future burden of AMR cannot be evaluated in isolation from climate change. Integrated modeling that combines climate projections with AMR burden estimates using metrics such as disability-adjusted life years (DALYs) and economic costing will be essential for developing effective policies and prioritizing resources to mitigate this converging crisis.

## 5. Post-Pandemic Scenarios for Climate and AMR

The emergence of Severe Acute Respiratory Syndrome Coronavirus 2 (SARS-CoV-2) from a wildlife wet market in Wuhan, China, in 2019, and its subsequent global spread, resulted in an estimated 197 million confirmed cases and over 4 million fatalities, fundamentally altering global public health priorities [74]. While some argued such a pandemic was unpredictable, its underlying drivers, i.e., zoonotic spillover, rapid global transmission, and severe economic disruption prompted a critical re-examination of the interconnected risks facing a highly connected world. The COVID-19 pandemic starkly revealed how global crises can converge: existing community vulnerabilities, exacerbated by both the pandemic and the ongoing effects of climate change, led to elevated rates of disease and mortality [75,76]. In 2020 alone, approximately 92 of the 132 documented extreme weather events posed direct risks to overburdened public health systems. For instance, droughts in Zimbabwe left millions without adequate food or clean water, while power outages in hydropower-dependent southern African nations critically hampered pandemic coordination efforts. Furthermore, an estimated 437.1 million people in vulnerable groups were exposed to extreme heat in 2020 [77], and in Australia, severe air pollution from an intense wildfire season likely worsened COVID-19 morbidity [78]. The pandemic thus serves as a potent case study in systemic risk, illustrating how climate events can amplify public health emergencies and overwhelm societal capacity to respond.

In a globalized world, the threats posed by climate change and emerging infectious diseases are systemic, interconnected, and increasingly recurrent. Their historically stable patterns of probability and distribution are shifting rapidly, rendering them nonstationary risks that are difficult to predict or control using past data [79]. When critical thresholds are crossed such as key surface temperature limits in climate systems or hospital capacity during disease outbreaks, the socioeconomic consequences become severe and widespread. Both climate change and pandemics like COVID-19 act as risk multipliers, exposing and intensifying preexisting vulnerabilities within societal and infrastructural systems. Effectively addressing these challenges requires a fundamental shift from short-term performance optimization toward building long-term, multi-dimensional resilience [80].

The COVID-19 pandemic starkly illustrated these interactions, particularly in relation to AMR. Although COVID-19 is a viral illness, a significant number of hospitalized patients developed secondary bacterial pneumonia, leading to widespread antibiotic use [81]. Secondary bacterial infections were present in nearly half of COVID-19 cases, yet only 8% of patients with bacterial co-infections actually required antibiotics, according to the Infectious Diseases Society of America (IDSA) [82]. A meta-analysis by Langford et al. further highlighted this issue, showing that 74.6% of COVID-19 patients received antibiotics inappropriately [83]. This underscores the urgent need for rapid diagnostic tools to distinguish viral from bacterial infections and for stricter antibiotic stewardship to reduce AMR-related morbidity and mortality. It also emphasizes the broader necessity for more resilient health systems and novel anti-infective therapies [84].

The convergence of climate and health crises further deepens these vulnerabilities. The Lancet Countdown on Health and Climate Change 2022 report noted that heatwaves intensified by climate change disproportionately endanger vulnerable populations such as the elderly and young children, a risk compounded by the COVID-19 pandemic [85]. Meanwhile, climate change continues to alter the transmission patterns of infectious diseases, increasing the incidence of zoonotic and vector-borne outbreaks and raising the threat of hospital-acquired infections [86]. The emergence of new diseases, overlapping with existing ones, strains resources and may lead to shortages in essentials such as personal protective equipment (PPE), thereby further amplifying the risk of AMR transmission [87].

Additionally, environmental factors worsened health outcomes during the pandemic. Climate change and associated phenomena including extreme heat and particulate air pollution elevate cardiovascular morbidity, which is a known risk factor for severe COVID-19 outcomes [88]. While research continues to clarify the nature of these relationships, it is evident that climate change influenced both the course and impact of the COVID-19 pandemic. Moving forward, integrated policy and preparedness strategies that simultaneously address climate change, AMR, and pandemic threats are essential for reducing future systemic risks.

On the other hand, the COVID-19 pandemic also yielded unintended environmental benefits, offering a glimpse into the potential for rapid planetary recovery under reduced anthropogenic pressure. The global lockdowns, implemented to curb the virus’s spread, inadvertently led to a significant, albeit temporary, improvement in environmental conditions. Notably, Venice, Italy, experienced a dramatic decline in tourism, resulting in unusually clear waterways due to the absence of boat traffic and sediment disturbance. Concurrently, efforts to mitigate viral transmission led to enhanced water treatment protocols; for example, wastewater facilities in China intensified disinfection measures, primarily through chlorination, to inactivate the virus [88,89].

The most profound environmental impact was observed in air quality. Research indicates that lockdown measures caused a substantial reduction in atmospheric pollutants. Major cities in China, Italy, France, and Spain witnessed a 20–30% decline in nitrogen dioxide (NO_2_) emissions, while a similar reduction of approximately 30% was recorded in the United States. Overall, China experienced an 11.4% improvement in air quality compared to the previous year. Similar trends were observed globally: Brazil reported drastic reductions in NO, NO_2_, and CO concentrations during its partial lockdown, and India saw a 12.33% reduction in levels of PM10, PM2.5, SO_2_, NO_2_, CO, O_3_, and NH_3_ [90]. This notable but temporary enhancement in air quality underscores the significant health benefits that could be achieved through a sustained transition to cleaner energy and transportation systems.

## 6. Impact of AMR Along with Climate Change on Food Security

The global food system, which provides sustenance for the most of the world’s population and supports the livelihoods of over 200 million people, is an intricate network involving production, transportation, processing, packaging, storage, retail, and consumption. Since 1961, per capita food production has increased by more than 30%, a achievement made possible by an 800% rise in the use of nitrogen fertilizers and a more than 100% increase in agricultural water consumption. However, these gains in productivity have come at significant environmental cost, and concerns about long-term global food security remain acute for the coming decades [91].

**Figure 5:**
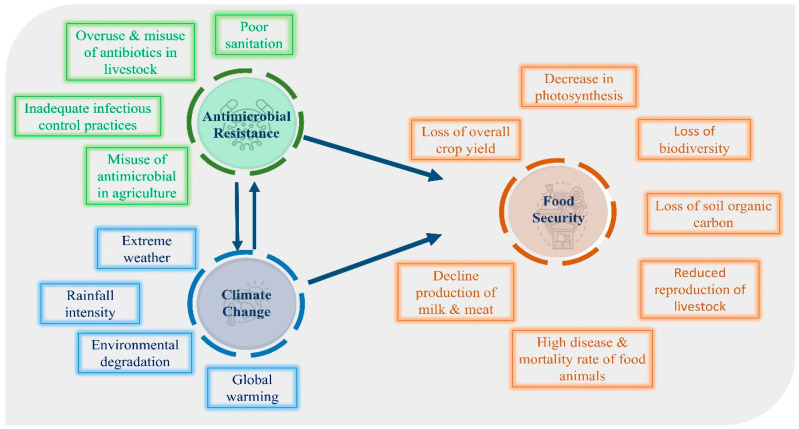
Antimicrobial resistance and climate change are interconnected, affecting food security and climatic factors like temperature, heat, wind, and rain. This synergistic effect compromises both qualitatively and quantitatively the food system.

Climate change and AMR now converge as major threats to this system, each exacerbating the other to create compound risks to public health and economic stability (Figure 5). Climate change facilitates the spread of vector-borne, waterborne, and foodborne diseases such as cholera and Zika virus through rising temperatures, extreme weather, and altered precipitation patterns. Simultaneously, AMR undermines our ability to treat these and other infections, threatening to trigger widespread health and financial crises. It is projected that climate change will contribute to over 250,000 additional deaths annually by 2030, while AMR could precipitate a global financial crisis, severely disrupting trade and productivity [92].

Population growth, especially in low- and middle-income countries where access to nutritious food is already unreliable, continues to increase the demand on the global food system [93]. This pressure often leads to intensified agricultural practices, including the inappropriate use of antimicrobials in livestock and crops, which further drives AMR. To mitigate these interconnected risks, urgent and coherent action is needed across government and private sectors. Policies must be integrated to promote sustainable agriculture, strengthen veterinary and human health systems, and ensure that efforts to combat climate change and AMR are aligned in protecting food security.

Climate change poses a significant threat to nutritional quality, plant and animal health, and food safety [94]. However, the relationship between climate change and food safety involves numerous risks and variables, resulting in considerable uncertainty. It is widely thought that climate change severely impacts human, plant, animal, and environmental systems, potentially exacerbating a range of foodborne illnesses [95]. According to the Fourth Report of the United Nations Intergovernmental Panel on Climate Change (IPCC), extreme weather events, rising atmospheric CO_2_ levels, altered precipitation patterns, and increasing temperatures are projected to critically endanger food safety [96]. To address this, the “Climate Change and Emerging Risks for Food Safety” project that is a multidisciplinary network of international and intergovernmental experts was established to identify climate change-related emerging risks to food safety [97]. This initiative has determined that climate change affects the occurrence, persistence, dominance, and toxicity of marine and freshwater algal blooms, as well as bacteria, fungi, viruses, parasites, and pathogenic vectors affecting plants and animals. Altered climatic factors may affect the sources and modes of transmission, growth, survival, and ecology of foodborne pathogens. For instance, temperature shifts can alter pathogens’ reproductive periods, potentially introducing new hazards or increasing host susceptibility to existing ones [94].

The excessive use of antibiotics in livestock, aquaculture, and crop production has contributed to AMR as a developing global crisis. Over 73% of antibiotics are used in the meat industry, promoting the development of antimicrobial-resistant bacteria in food-producing animals. Global antibiotic usage in livestock is expected to rise by 67% by 2030 [98]. Major food-borne and waterborne pathogens including *V. cholerae*, *Campylobacter* spp., *L. monocytogenes*, *Salmonella* spp., *E. coli*, and *Arcobacter* spp. are now frequently antibiotic-resistant. This resistance may be further amplified by climate change; for example, Johnsen and Kroer observed that warmer temperatures enhanced plasmid-mediated gene transfer between *E. coli* and *Pseudomonas putida*, suggesting that higher temperatures could facilitate the horizontal transmission of resistance genes [50]. A similar trend of resistance to antifungal treatments is emerging among fungi. As climate change-driven warmer temperatures increase the global prevalence of microbial diseases, a corresponding rise in antimicrobial resistance is anticipated. The Food and Agriculture Organization (FAO) has reported that rising average ambient temperatures in certain regions are already linked to increased antimicrobial resistance [99].

**Figure 6:**
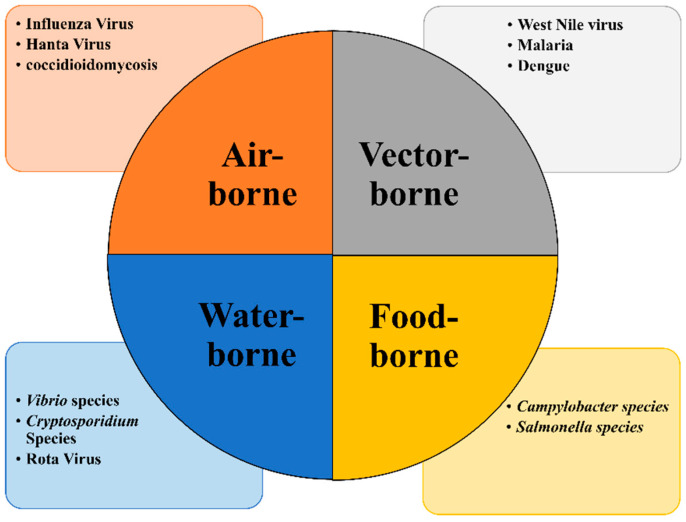
Pathogen and diseases in relation to climate change.

Both antimicrobial resistance and climate change critically endanger global food security and production, thereby undermining sustainable agricultural practices. A vast number of plant-specific microorganisms and the diseases they cause threaten the quality and quantity of the food chain. Certain infections, including food-borne, water-borne, and vector-borne diseases, respond differently to environmental conditions. Agro-adapted diseases, whose spread is directly influenced by agriculture and human activity, are becoming more resistant and virulent due to limited treatment options [100]. The interconnectedness of pathogens, infectious diseases, environmental factors, and climate change is illustrated in Figure 6.

Rising temperatures and shifting environmental conditions are expanding the geographical ranges of pests and pathogens. To combat these threats, agriculture has increasingly relied on antibiotics such as streptomycin and oxytetracycline. This practice results in higher drug residues on plant-based commodities that enter the food chain. Furthermore, the use of contaminated water for irrigation introduces additional foodborne pathogens and antimicrobial residues into fresh produce [95]. Beyond food safety, climate change negatively impacts food and water security, decreasing agricultural productivity, undermining farmers livelihoods, and exacerbating poverty [101]. This is particularly critical in developing countries, where agricultural systems are vital to both household income and national economies [102]. Projected temperature increases pose a significant risk to global agricultural output, adversely affecting future growth [103].

In this context of climate change and rising antimicrobial resistance, the application of beneficial plant- and animal-associated microorganisms is gaining recognition as a more sustainable approach to supporting food production. However, agricultural practices also contribute to climate feedback loops. For instance, methanogenic bacteria in artificial and natural anaerobic environments generate the potent greenhouse gas methane (CH_4_) [104]. Unsustainable practices, including the excessive use of fertilizers, alter vital biogeochemical cycles such as carbon and nitrogen. The burning of fossil fuels for agriculture further disrupts these systems. While rhizobacteria in plant roots can convert the greenhouse gas N_2_O into inert N_2_, disturbances to these microbial communities can impair this process. The disruption of soil microbiota’s nitrate reductase activity increases atmospheric N_2_O levels, creating a reinforcing cycle that further contributes to climate change [105].

Food security exists when a population, household, or individual has consistent physical, social, and economic access to sufficient, safe, and nutritious food. As defined by the FAO, this concept rests on four pillars: food availability, access, utilization (through adequate diet, clean water, sanitation, and healthcare), and stability over time [106]. These pillars are profoundly threatened by the interconnected crises of climate change and AMR, which jeopardize the health and well-being of millions worldwide.

A range of global organizations—including agrifood companies, the World Bank, the World Trade Organization (WTO), the FAO, and social movements exert varying degrees of influence to shape the global food system according to their respective policy agendas, sales targets, or objectives [107]. The FAO already monitors key sustainability metrics, including soil nutrient budgets, livestock trends, and indicators related to land use, fertilizer, and pesticide application [108]. To safeguard food security, it is imperative to prioritize sustainable farming practices and promote the prudent use of antibiotics in both human and animal health. Furthermore, we must invest in research for alternative solutions and implement robust policies that mitigate the impacts of climate change.

**Figure 7:**
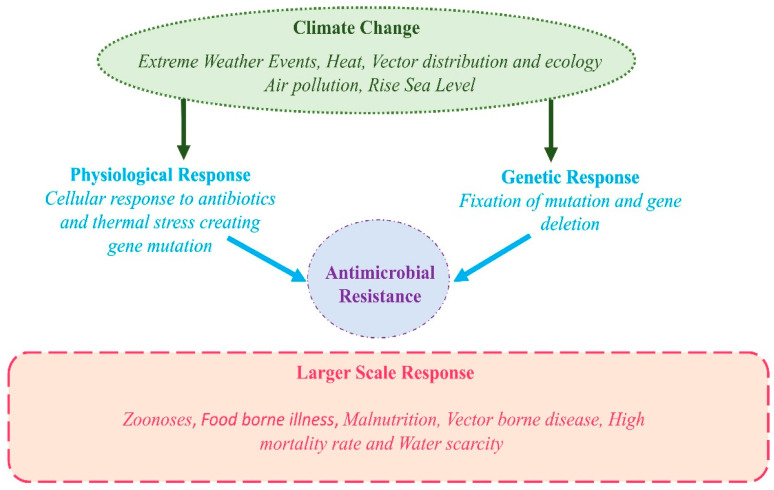
Antimicrobial Resistance in Response to Climate Change.

Climate change promotes AMR through a multitude of interrelated mechanisms (Figure 7). Rising temperatures directly exacerbate antibiotic resistance by facilitating the geographic spread of pathogens and accelerating molecular mechanisms such as horizontal gene transfer [71]. Furthermore, extreme weather events, notably floods, increase human exposure to contaminated water sources; this has been associated with epidemics of resistant *Vibrio cholerae*, as witnessed in Yemen [32]. Similarly, ocean warming and changes in salinity promote the survival and spread of resistant pathogens like *Vibrio vulnificus* and *Candida auris* [109]. Consequently, adopting a One Health strategy is imperative to align efforts toward simultaneously safeguarding human, animal, and environmental health. The established correlation between global warming and the spread of infectious diseases, particularly those of zoonotic origin, underscores the critical need for a cooperative, multisectoral, and transdisciplinary approach. This method acknowledges the interconnected health of humans, animals, and ecosystems, demanding coordinated action at local, regional, national, and global levels [110].

### Interventions and Recommendations at the Human–Animal–Environment Interface

**Figure 8:**
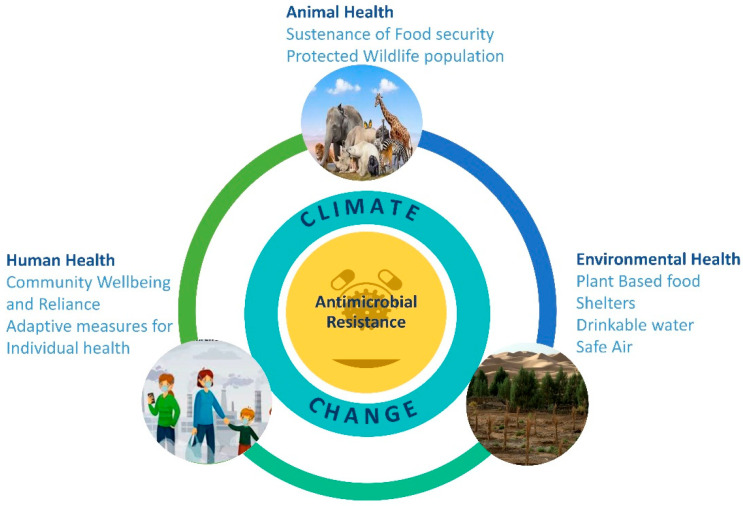
One Health approach incorporated with climate change and AMR.

The adoption of a One Health paradigm enables the implementation of multidisciplinary and transdisciplinary strategies that transcend the traditional boundaries of public health and environmental sustainability. This approach, which simultaneously addresses AMR and climate change, offers significant advantages by enhancing food security, sustaining animal food sources, improving livestock systems, and promoting environmental hygiene [111] (Figure 8). Furthermore, it facilitates the establishment of integrated global syndromic surveillance and response systems. Such a framework is critical for interpreting and applying findings from a unified perspective, which is essential for preventing more severe global catastrophes [112]. By explicitly acknowledging the interconnectedness of human, animal, and environmental health, the One Health concept provides a holistic and unified framework to mitigate the dual threats of pandemics and climate change, thereby safeguarding the well-being of both humanity and the planet [80].

Global One Health initiatives are dedicated to advancing global health security through the establishment of strategic policies and programs. By fostering collaboration across the human, animal, and environmental health sectors, these initiatives play a pivotal role in supporting the achievement of international health legislation and the Sustainable Development Goals (SDGs) [113]. They facilitate the exchange of information, resources, and best practices to address pervasive health risks that transcend national borders. Through collaborative efforts and partnerships, these activities are indispensable for safeguarding public health and advancing sustainable development on a global scale [114].

A landmark step in this collaboration was the April 2010 tripartite agreement between the WHO, the FAO, and the WOAH to address critical issues including antimicrobial resistance, rabies, tuberculosis, and the Middle East respiratory syndrome coronavirus. This agreement established a long-term strategic direction for international cooperation, aiming to coordinate global health efforts at the human–animal–ecosystem interface [115]. Significant advancement occurred on 17 March 2022, when the United Nations Environment Program (UNEP) joined the alliance, forming a new quadripartite agreement. This expansion marks a turning point in the One Health approach, as it formally acknowledges the critical role of the environment in health outcomes [116]. The directors general of these organizations signed a memorandum of understanding, committing to enhanced collaboration for the health of people, animals, plants, and the environment. This partnership not only creates a robust legal framework for addressing challenges at the nexus of humans, animals, and ecosystems but also strengthens health services at global, regional, national, and local levels. By promoting cooperation, knowledge exchange, and resource-sharing, this quadripartite enables a more effective response to cross-border health challenges, such as drug resistance and emerging infectious diseases. The ultimate goal of this alliance is to enhance global health security and promote sustainable development by comprehensively addressing these interconnected health threats [117].

In direct response to global calls for a sustainable and preventative health approach, the Quadripartite has developed the One Health Joint Plan of Action [OH JPA (2022–2026)] to operationalize the One Health strategy and prevent future pandemics [118]. An overview of the theory of change for the OH JPA is presented in Table 2. The plan’s six action tracks are guided by four cross-cutting principles: (i) adopting systems thinking, (ii) fostering advocacy and public–private partnerships (PPPs), (iii) improving governance and legal frameworks, and (iv) utilizing the traditional knowledge of Indigenous Peoples and local communities where appropriate. The purpose of these principles is to address common underlying challenges and create synergies across the action tracks.

The Lancet Countdown initiative is dedicated to monitoring the health impacts of climate change, tracking progress in health adaptation and resilience, and quantifying the health co-benefits of mitigation efforts [119]. An integrated One Health strategy is critical to address these climate-associated health challenges, as it can unify diverse scientific disciplines, policy-making bodies, and local knowledge systems. In areas such as monitoring antimicrobial resistance and facilitating climate adaptation, One Health initiatives have been shown to surpass traditional, siloed approaches to public and animal health. However, significant gaps remain, including a lack of cooperation across different sectors and insufficient regulatory frameworks to effectively govern professional services [120].

One Health aims to achieve optimal health outcomes for people, animals, and the environment through the collaborative efforts of multiple disciplines and sectors at local, national, and global levels. This approach fosters innovation to address both acute and long-term health threats, enhances communication, cultivates a new generation of systems-thinking professionals, improves surveillance, shortens response times, and delivers economic and public health benefits [121]. Specifically, a One Health framework provides an integrated structure for combating AMR in the context of climate change by coordinating interventions across human, animal, and environmental domains. On the human health front, improving antibiotic stewardship in clinical settings is a critical component of AMR mitigation, as inappropriate prescribing accelerates resistance across diverse pathogens [122]. Expanding immunization programs also plays a vital role by preventing infections that would otherwise require antimicrobial treatment, thereby reducing unnecessary antibiotic use [123]. Furthermore, enhanced surveillance systems, such as the WHO Global Antimicrobial Resistance and Use Surveillance System (GLASS), enable real-time monitoring of resistance patterns. This capability allows for earlier interventions and guides the development of evidence-based policy [124].

The routine systemic use of antibiotics in animal production for growth promotion and disease prevention is a primary driver of global antimicrobial consumption. Estimates indicate such use is projected to rise by 67% by 2030, particularly in low- and middle-income countries [125]. Curbing this trend requires stringent regulation alongside the promotion of sustainable alternatives. For instance, livestock vaccinations and probiotics have proven effective in reducing infection rates without increasing selective pressure for resistance [126]. Furthermore, enhanced farm biosecurity encompassing improved hygiene practices, reduced animal crowding, and robust disease surveillance minimizes dependence on antibiotics and mitigates the risk of resistant bacteria transferring from animals to humans.

The environmental dimension of this issue is equally critical. Antibiotic residues from healthcare, agriculture, and pharmaceutical manufacturing routinely enter waterways and soils, creating hotspots for resistance gene evolution. Upgrading wastewater treatment facilities with advanced filtration technologies is essential to reduce the load of resistant bacteria and antibiotic residues entering ecosystems [127]. Stricter regulations governing antibiotic disposal and agricultural runoff are needed to prevent environmental contamination, while surveillance of AMR in soil and aquatic environments can provide an early warning system for emerging resistance reservoirs [128]. A One Health strategy moves beyond abstract conceptualization by integrating interventions across all three domains simultaneously. This ensures coordinated, measurable actions are taken to prevent the proliferation and spread of resistance in an era of climate upheaval.

## 7. Diverse Actions: Global Strategies for Climate Change and AMR

Climate change and AMR represent two of the most pressing global public health crises of our time [129]. MR is primarily driven by the misuse and overuse of antimicrobials across human health, animal health, and agricultural sectors. Critically, climate change acts as a threat multiplier, exacerbating the spread and impact of AMR and thereby intensifying the overall risk to public health. Adopting the One Health paradigm offers a comprehensive framework for addressing these interconnected challenges, as it recognizes the inherent interdependence of environmental, animal, and human health [54]. By understanding the complex relationships between human activities, environmental factors, and health outcomes, we can develop more effective strategies to mitigate the combined effects of AMR and climate change on global health [130].

While the One Health approach provides an essential integrative framework, it is also critical to acknowledge the concerted efforts of global organizations actively combating these threats. The World Health Organization (WHO) recognized AMR as a critical public health threat, issuing a stark warning in 2014. This declaration emphasized the urgent need for a coordinated global response to combat drug-resistant infections. In response, the World Health Assembly endorsed the Global Action Plan (GAP) on AMR, urging all member states to develop and implement national action plans aligned with its objectives by May 2017 [131]. The core objectives of this pivotal plan are outlined in Table 3. This landmark initiative established a foundation for international collaboration by highlighting the value of surveillance, stewardship, and innovation in countering the rise of resistant pathogens.

Global efforts guided by this framework have promoted actions such as the prudent use of antibiotics to reduce the incidence and transmission of AMR. Complementing these efforts, agencies like the US Food and Drug Administration have developed specific protocols for monitoring and evaluating AMR outbreaks. In the ongoing battle against AMR, enhancing rapid diagnostic capabilities is paramount. This is especially crucial in resource-limited settings where conventional microbiological methods are often unavailable or slow. Advancements in new genetic screening technologies can help overcome this barrier by enabling precise pathogen identification and guiding targeted antimicrobial therapy [132].

**Figure 9:**
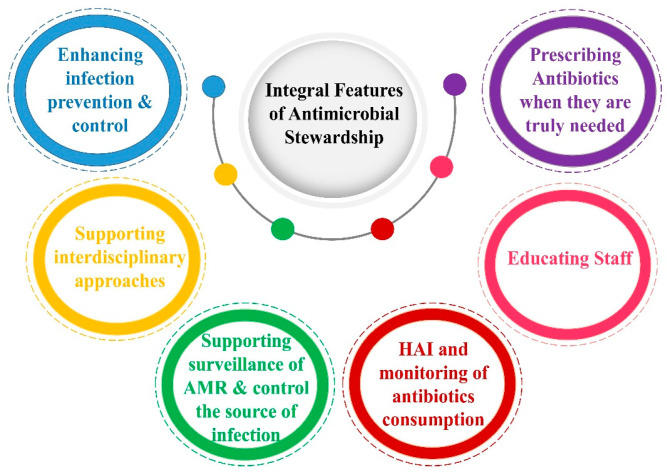
Integral Features of Antimicrobial Stewardship Program to treat infections and reduce antimicrobial resistance.

Antimicrobial Stewardship (AS) is a multidisciplinary approach essential for improving patient treatment outcomes and safety by curbing the development of AMR. It coalesces expertise from diverse fields, including healthcare leaders, microbiologists, infectious disease specialists, physicians, nurses, farmers, veterinarians, IT experts, and clinical pharmacists [133] (Figure 9). Rooted in the “One Health” concept, this approach arises from a multifaceted organizational strategy that integrates animal, human, and environmental health. Within this integrated framework, Antimicrobial Stewardship is one of three critical pillars for strengthening health systems, alongside patient safety, medicine safety, and infection prevention and control (IPC) [134]. The success of an Antimicrobial Stewardship Program (ASP) is inherently tied to its inclusivity, as the scope of each component is too vast for any single discipline to manage alone. In this endeavor, healthcare epidemiologists and infection preventionists are indispensable for the effective implementation and sustainability of ASPs [133]. By optimizing antibiotic use, AS plays a direct role in controlling AMR. When combined with the other two pillars and ensuring a sufficient supply of quality-assured medications and robust AMR surveillance, these elements form a foundation for equitable, high-quality healthcare that advances the goal of universal health coverage.

**Figure 10:**
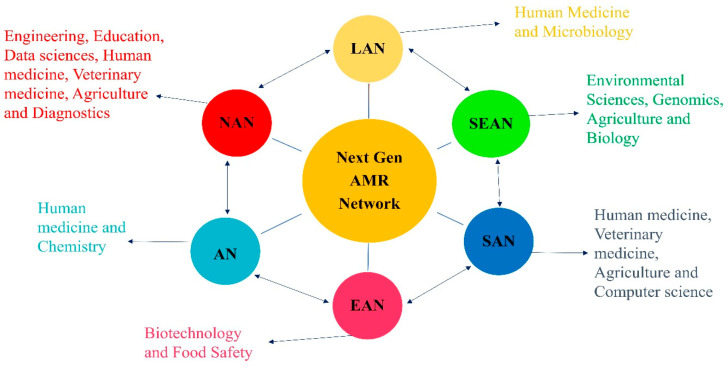
Next-generation AMR network. NAN = North American Network; LAN = Latin American Network; SEAN = Southeast Asian Network; SAN = South Asian Network; EAN = East Asian Network; AN = Asian Network.

Addressing the global challenge of AMR necessitates unprecedented international collaboration. A global network-to-network (NTN) approach is required to discover optimal solutions, accelerate scientific research, and effectively integrate these advancements into existing health systems. This next-generation AMR network will equip current and future researchers with the framework necessary for large-scale, multidisciplinary, and multinational cooperation. Its membership must be truly representative, encompassing high-income countries (HICs) and LMICs from diverse continents to capture a wide range of economic contexts, geographical locations, climatic conditions, cultures, and resources. By leveraging existing discipline-based networks, the proposed AMR NTN system would establish a transdisciplinary research pathway dedicated to AMR innovation and discovery [135]. As illustrated in Figure 10, this transdisciplinary network will forge new connections between regional initiatives while effectively harnessing deep discipline-specific and localized knowledge.

Scientific networks encompassing multiple institutions are actively engaged in combating AMR through the development of rapid diagnostic tools for bacterial and viral pathogens. A key example is the Global Alliance for Rapid Diagnostics (GARD). Established in 2016, GARD seeks to create internationally deployable, inexpensive biosensing technologies for the early detection of infectious diseases, leveraging advancements in nanotechnology (https://www.egr.msu.edu/alocilja/global-alliance-rapid-diagnostics-gard, accessed on 30 June 2025). To achieve this, GARD has established six regional networks focused on creating low-cost, user-friendly diagnostic solutions capable of storage at room temperature. This collaboration has yielded significant outcomes, including joint publications, faculty exchanges, symposiums, and the education of a new generation of scientists in interdisciplinary approaches to AMR problem-solving. Building on this foundation, and guided by the One Health approach, the next generation of AMR networks can concentrate on advancing a transdisciplinary paradigm known as “Sustainable Health through Innovation (SHI).” This paradigm aims to leverage technological advancements to develop durable, long-term solutions for AMR [135]. In parallel, many researchers are focusing on another strategic pillar: the development of antimicrobial substitutes to combat resistant pathogens [136] (Table 4).

Global strategies for addressing the threat of climate change are broadly categorized into two approaches: (i) mitigation, involving actions to reduce greenhouse gas (GHG) emissions, and (ii) adaptation, entailing adjustments in human behavior and systems to accommodate unavoidable climatic changes [147]. While mitigation is crucial for long-term economic and environmental stability, adaptation often has a more direct and immediate impact on managing the health consequences of extreme weather events, such as floods.

**Figure 11:**
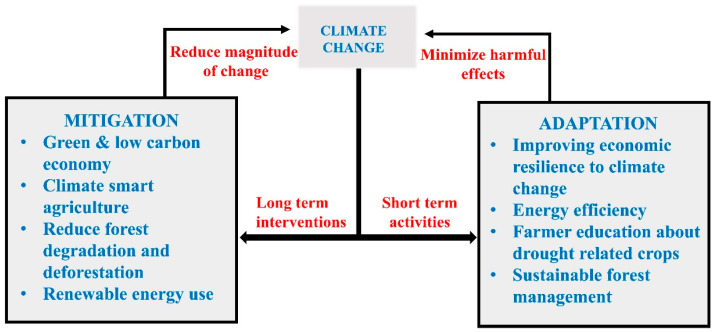
Adaptation and mitigation strategies for climate change.

Effective adaptation strategies, such as reforestation, improved drainage systems, and enhanced sanitation, can directly assist in combating climate-sensitive infectious diseases, particularly in African nations. These actions align with those suggested by the UNEP in 2013 to slow the impacts of climate change [148]. The public health response to climate change is characterized by two predominant schools of thought. The first posits that the effects will inevitably intensify and must be countered by increased investment in health-related infrastructure [149]. The second perspective emphasizes that climate change not only poses direct physical threats but also risks weakening public health systems themselves, making resilience a critical priority. In this context, accurate weather forecasting is essential for public preparedness. For instance, early warnings of severe droughts and tropical cyclones issued by the Pacific ENSO Application Center (PEAC) during the 1997–1998 El Niño period were instrumental in reducing hospital admissions for diseases like dengue, malaria, and diarrhea [150]. However, scientists express concerns regarding the variable effectiveness of these adaptation and mitigation techniques across different geographic and economic contexts. Ultimately, implementing these strategies requires integrated policy actions across primary sectors, including agriculture, forestry, industry, transportation, and land use. Key adaptation and mitigation strategies are illustrated in Figure 11.

The health sector, which has now surpassed shipping and aviation, is responsible for 4% of global greenhouse gas emissions [151]. To limit warming to 1.5 °C without relying on carbon removal technologies, all sectors including healthcare must achieve carbon neutrality by 2050, exceeding the initial ambitions of the Paris Agreement [152]. Emissions from the health sector are primarily influenced by three factors: the demand for health services, the carbon intensity of the broader economy, and the emissions profile of the energy grid.

Significant emission reductions in healthcare are both achievable and compatible with improving population health. For instance, the UK National Health Service (NHS) has committed to an 80% reduction in emissions by 2050. Realizing such goals requires substantial investment in energy efficiency and strong advocacy from within the health industry itself [153]. The sector can lead by example, implementing mitigation strategies that also yield public health benefits. This includes promoting healthy diets, walking, and cycling to simultaneously reduce environmental pollution and curb obesity. Furthermore, high-income health systems have a responsibility to support the sustainable development of robust healthcare infrastructure in lower-income nations.

The COVID-19 pandemic caused a temporary decline in greenhouse gas emissions, providing a unique opportunity. Health systems should leverage this moment to advocate for and embed lasting environmental conservation practices as they rebuild. Universal implementation of climate change mitigation in healthcare is essential to meet widely accepted international goals and advance the sector’s environmental sustainability [154]. It is crucial to note that the COVID-19 pandemic has also significantly influenced AMR trends. Widespread empirical antibiotic use in COVID-19 patients often without confirmed bacterial co-infection contributed to elevated resistance rates globally. Simultaneously, surveillance disruptions and diverted funding severely hindered AMR monitoring and containment efforts. These pandemic-related shifts must be carefully considered when assessing the recent global AMR burden, as they represent both a critical challenge and a vital lesson for future integrated pandemic-AMR preparedness planning.

Adaptation and mitigation initiatives demand careful consideration and coordinated action at both national and international levels. Over recent decades, climate change has escalated into an urgent global crisis, and the world’s future economic and social advancement is now fundamentally dependent on our preparedness for its effects. To effectively mitigate and adapt to these impacts, nations must align their efforts with overarching global strategies and policies [68]. The scope of these global strategies has expanded, yet their execution remains highly diverse and uneven. This disparity is evident in the global response to AMR, a crisis exacerbated by climate change. For instance, as of 2022, over 170 countries have developed National Action Plans (NAPs) on AMR; however, less than 20% have reported securing dedicated financing streams for their implementation. Progress is further measured by key indicators such as the proportion of countries implementing routine AMR surveillance as only 71% participate in the Global Antimicrobial Resistance and Use Surveillance System (GLASS) and the establishment of functional antimicrobial stewardship programs in hospitals, which remains below 40% globally. Ultimately, insufficient financing and inequitable implementation persist as the primary impediments to achieving meaningful progress.

## 8. The Potential Role of AI in Tackling Interconnected Global Challenges

The correlation between rising global temperatures and increased infection rates is becoming increasingly evident. However, the intricate relationship between AMR and climate change remains incompletely understood. The application of artificial intelligence (AI) and machine learning (ML) offers a powerful, alternative lens through which to analyze this complex interplay. Despite the promising potential of these technologies, published research demonstrating their successful integration remains limited. This scarcity is primarily due to the significant challenges inherent in integrating disparate, multi-source data and the current absence of robust, standardized validation methodologies [155].

Current initiatives at the nexus of climate change and AMR predominantly focus on surveillance and forecasting, which are critical for implementing preventative public health measures and tracking disease spread [156]. Ecological niche models and ML algorithms, for instance, are being used to predict the spatial movement of fly vector species. These models indicate that climate change elevates the risk of leishmaniasis spread into regions such as North Texas and northern Mexico. Similarly, an AI-driven approach identified temperature-related meteorological variables as the strongest predictors for weekly infectious diarrhea episodes, while average weekly rainfall data proved to be a less reliable indicator [157].

The utility of machine learning extends beyond public health into climate science itself. ML techniques are increasingly advocated to unravel new insights into the Earth’s system processes and their interactions with biological cycles. Concurrently, in the fight against AMR, AI-based methods such as those leveraging Whole-Genome Sequencing data for Antimicrobial Susceptibility Testing (AST) are being developed to rapidly and accurately identify resistance patterns, which is particularly valuable in treating complex, chronic infections [158]. The efficacy of these advanced techniques is fundamentally constrained by the quality and comprehensiveness of the underlying data. While numerous public AMR databases exist, their utility for training robust AI prediction models is often hampered by irregular updates and a critical lack of consistent data standardization [159]. Although current AI technologies exhibit limitations, they hold considerable promises for addressing AMR. Future research should focus on overcoming data scarcity challenges through advanced methods like unsupervised learning for the automatic annotation of unlabeled data, transfer learning, and few-shot learning to broaden the applicability of AI in combating AMR [160,161].

In summary, AI has become a ubiquitous term with clearly defined applications in both climate analysis and AMR interpretation. While machine learning will be instrumental in exposing hidden patterns and enhancing predictive models for climate and AMR, AI will leverage these insights to support decision-making and ensure safety in challenging environments. The embrace of machine learning is poised to generate refined climate projections for higher greenhouse gas concentrations, thereby contributing to the step-change in ambition required for effective climate policy [162].

## 9. Conclusions

Climate change and AMR represent two of the most pressing global threats to human and planetary health, yet both can be restrained through the implementation of evidence-based, integrated policies and practices. Embracing the One Health initiative which acknowledges the inextricable links between human, animal, and environmental health is fundamental to mitigating this dual crisis.

**Figure 12:**
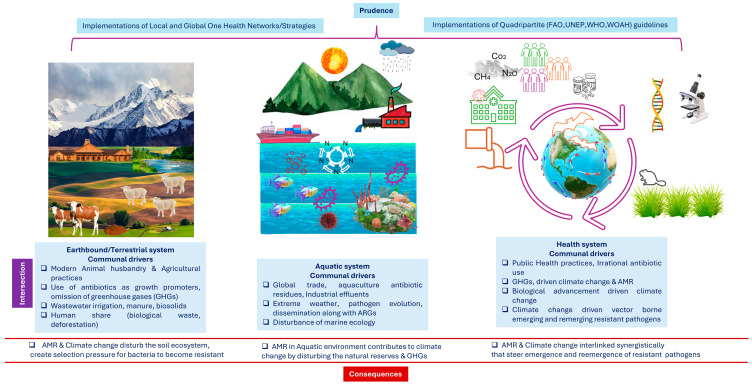
Schematic illustration, depicting various intersections between climate change and AMR along with deleterious consequences.

The negative consequences of climate change and AMR disproportionately affect vulnerable populations, including infants and those over 65, a disparity that has been exacerbated by the COVID-19 pandemic. The climate crisis amplifies the risk of emerging infectious diseases and AMR, impacting disease transmission patterns and straining global economies. The protracted cause-and-effect chain of these issues means that the unsustainable use of carbon and antibiotics today imposes a severe burden on future generations. Global trajectories of both climate change and AMR are profoundly influenced by population dynamics and trade, and their intersections drive severe consequences across terrestrial, aquatic, and human health systems worldwide (Figure 12).

Amid and beyond the COVID-19 pandemic, it is critical to reinforce and evaluate antimicrobial stewardship guidelines to counteract pandemic-driven AMR. Empowering healthcare personnel and the public through education and adequate resources is essential. Effective public health initiatives require unprecedented collaboration across environmental, animal, and human health sectors, as championed by the quadripartite (FAO, WHO, UNEP, WOAH) alliance. Planetary health emerges as an exciting multidisciplinary framework to develop alternative solutions and build a resilient future.

The intricate connection between AMR and climate change demands greater focus and deeper investigation. To fully elucidate the pathways and quantify the impacts of these connections, future research must leverage localized data on weather patterns, resistance events, infectious disease incidence, and antibiotic usage. Making such investigations a reality requires dedicated funding and open access to scientific data. Moving forward, research should employ descriptive summaries and systematic literature reviews, incorporate burden-of-disease models, carefully distinguish correlation from causation, and prioritize high-impact case studies, such as the spread of mcr-mediated resistance to last-resort antibiotics [163].

In conclusion, addressing the interconnected root causes and implementing integrated strategies for AMR and climate change is not merely an option but a professional and ethical responsibility. Such efforts are essential to safeguard patient health, promote health equity, and foster economic resilience for generations to come. By combining mechanistic insights, epidemiological surveillance, and robust global strategies, we can minimize the devastating worldwide impact of these twin threats.

## Figures and Tables

**Table 1:** Overview of the Relationship between Climate Change and Infectious Diseases.

Microorganism	Disease	Role of Climate Change	Reference
*Leptospira* spp.	Leptospirosis	Heavy rainfall and floodwater are significantly involved in the spread of water contaminated with resistant pathogens.	[29]
*Campylobacter* spp.	Waterborne infection	An increase in water system temperature helps these microbes survive longer.	[30]
Dengue virus	Dengue	Warmer temperatures led to rising spread of vectors	[31]
*Trypanosoma cruzi*	Chagas Disease	Warmer temperatures increased the propagation of vectors, even in winter.	[31]
*Schistosoma* spp.	Intestinal schistosomiasis	An increase in temperature can widen transmission areas for fluke to spread infection.	[32]
*Vibrio cholera*	Cholera	Natural catastrophes brought on by rising temperatures created an environment that was more favorable to microbes.	[33]
*Plasmodium falciparum*	Malaria	Rising temperatures and humidity can lead to greater transmissibility.	[34]
SARS-CoV-2	COVID-19	Droughts and rising aridity have caused bat migration and virus transmission.	[35]
*Salmonella* spp.	Typhoid Fever	Increased ambient temperature leads to a direct rise in replication rates.	[36]
*Candida auris*	Candidiasis	Gained thermo-tolerance and salinity tolerance on the wetland ecosystem.	[37]

**Table 2:** Overview of One Health Joint Plan of Action.

Pathways of Change	Action Tracks	Outcomes
Pathway 1 Policy Legislation Advocacy Financing	1—Enhancing One Health capacities to strengthen the health system	Improved health of humans, animals, plants, and the environment while identifying sustainable system-wide One Health solutions that allow our ecosystems to thrive in harmony
	2—Reducing the risk of emerging and re-emerging zoonotic pandemics and epidemics	Reduced risk and impact of health threats at the human–animal–plant–environment interface using a One Health approach efficiently, effectively, and equitably.
Pathway 2 Organizational development Implementation Sectoral integration	3—Controlling and eliminating zoonotic, neglected tropical, and vector-borne diseases	Effective collaboration and synergy to build advocacy and political will and to leverage investment for an evidence based One Health approach.
	4—Strengthening the assessment, management, and communication of food security risks	Improved coordination, communication, and alignment of One Health activities and capacity-building efforts, including in the provision of technical support, normative frameworks, research, education, and guidance.
Pathway 3 Data collection Evidence Knowledge	5—Curbing the silent pandemic of Antimicrobial Resistance	Strengthened cross-sectoral capacity to co-design and implement inclusive and equitable multilevel work plans and strategies in line with One Health principles.
	6—Integrating the environment into One Health	Improved and harmonized One Health tools, technologies and practices that integrate data and knowledge are developed, disseminated and utilized.

**Table 3:** Key Objectives of Global Action Plan.

Global Action Plan	
1	Increase awareness and knowledge of antimicrobial resistance through Training, communication, and education.
2	Improve knowledge and evidence bases through surveillance and research.
3	Reduce infection rate by implementing adequate sanitation, hygiene, and infection prevention measures.
4	Optimize the use of antimicrobial drugs in human and animal health.
5	Establish an economic case for ongoing investment in new drugs, diagnostic tools, vaccinations, and other treatments.

**Table 4:** Alternatives to Antibiotics.

Alternatives	Properties	Advantages	References
Vaccines	Easy to use Limit the onset of disease	Promote Specific Immunological Protection Preventing Bacterial and Viral Infections	[137]
Monoclonal Antibodies	Prophylactic action Pre-emptive approach	Long half-life Highly specific Do not disrupt normal flora	[138]
Probiotics	Prebiotic Symbiotic Competitive exclusion	Useful for commensal gut bacterial health. Prevent pathogen colonization	[139]
Phage Therapy	Narrow host spectrum Great diversity Bacteriolysis	Lytic activity is independent of antibiotic resistance Do not infect eukaryotic cells Found naturally in the environment	[136]
Engineered Phages	Safe and well-regulated Increase the utility of Phages for therapy	Applicable against a wide variety of host ranges Highly effective against biofilms	[140]
Predatory Bacteria	Alter and consume other bacteria.	Effective against biofilm Can access recalcitrant infection	[141]
Herbal Medicine	Phytochemicals	Efflux inhibitory activity against Gram-negative bacteria Biofilm inhibitors Quorum-sensing inhibitors	[142]
Nanoparticles	Low minimum inhibitory concentration (MIC) Unique physical and chemical properties	Can target multiple cellular pathways at once It can penetrate through the cell wall and kill bacteria Used to treat multiple drug-resistant bacteria	[136]
Bacterial cell wall hydrolysis (BCWH)	Lysozymes Autolysins Virolysins	Highly effective against antibiotic-resistant bacteria Safe and well understood Immunogenicity is not a concern for their effectiveness	[143]
CRISPR-based Antimicrobial	Highly specific Target Multiple drug-resistant bacteria	Can be tuned for a variety of antimicrobial applications Reversal of antibiotic usage Specificity towards pathogenic strains	[144]
Antimicrobial Peptides	Diverse activities range from antibacterial, antifungal, antiviral, anticancer, and anti-plasmodial to immunomodulation.	Not prone to resistance development. Broad-spectrum activity is an advantage, depending on the application	[145]
Synthetic mimics of antimicrobial peptides (SMAMPs)	Polymer or oligomers Cost-effective than AMPs	Ease of synthesis Not prone to resistance development Broad-spectrum activity is an advantage, depending on the application	[146]

## Data Availability

No new data were created or analyzed in this study.
